# PVDF-BaTiO_3_ Nanocomposite Inkjet Inks with Enhanced β-Phase Crystallinity for Printed Electronics

**DOI:** 10.3390/polym12102430

**Published:** 2020-10-21

**Authors:** Hamed Abdolmaleki, Shweta Agarwala

**Affiliations:** Department of Engineering, Aarhus University, 8200 Aarhus, Denmark; hamedabdolmaleki@eng.au.dk

**Keywords:** piezoelectric, PVDF, barium titanate, nanocomposites, printed electronics, inkjet printing, nanomaterial ink

## Abstract

Polyvinylidene difluoride (PVDF) and its copolymers are promising electroactive polymers showing outstanding ferroelectric, piezoelectric, and pyroelectric properties in comparison with other organic materials. They have shown promise for applications in flexible sensors, energy-harvesting transducers, electronic skins, and flexible memories due to their biocompatibility, high chemical stability, bending and stretching abilities. PVDF can crystallize at five different phases of α, β, γ, δ, and ε; however, ferro-, piezo-, and pyroelectric properties of this polymer only originate from polar phases of β and γ. In this research, we reported fabrication of PVDF inkjet inks with enhanced β-phase crystallinity by incorporating barium titanate nanoparticles (BaTiO_3_). BaTiO_3_ not only acts as a nucleating agent to induce β-phase crystallinity, but it also improves the electric properties of PVDF through synergistic a ferroelectric polarization effect. PVDF-BaTiO_3_ nanocomposite inkjet inks with different BaTiO_3_ concentrations were prepared by wet ball milling coupled with bath ultrasonication. It was observed that the sample with 5 w% of BaTiO_3_ had the highest β-phase crystallinity, while in higher ratios overall crystallinity deteriorated progressively, leading to more amorphous structures.

## 1. Introduction

Direct printing with nanomaterial inks has gained much interest lately due to its potential in fabricating flexible electronic components such as sensors [1], actuators [2], batteries [3], supercapacitors [4], transistors [5], etc. Generally, printing techniques can be classified into two categories, namely “contact” and “non-contact” printing. In contact printing, patterns are formed inside the printer (on engraved rollers or a stencil) and transferred directly onto the substrate; however, in non-contact techniques, the patterns are deposited onto the substrate through one or a series of computer-controlled nozzles. Flexography, gravure, offset, and screen printing are the most well-known contact printing techniques, while inkjet, aerosol jet, and electrohydrodynamic jet printing are the most prominent non-contact methods.

Nanomaterial inks used in inkjet printing play a crucial role in imparting functionality to the printed pattern. Hence, a lot of impetus has been put on building a library of new nanomaterial inks for flexible electronics. For satisfactory inkjet printing, inks have to meet certain fluid mechanical requirements, otherwise printing may suffer from satellite drops, splashing, the coffee-ring effect, and nozzle clogging. To quantify fluid mechanical requirements, Fromm et al. introduced a dimensionless *Z* value, which is defined by dividing the Reynold number (Re) number by the square root of the Weber (We) number [6].

According to Reis and Derby, the interval of 1 < *Z* < 10 is the inkjet-printable window [7], where the lower limit demonstrates the minimum *Z* value below which the drops cannot be ejected from the nozzles and the upper limit is the starting point of satellite drop formation. In other research, Jang et al. reported the interval of 4 < *Z* < 14 as the inkjet-printable range [8], which was more consistent with the results achieved in this research. Much focus has been put on synthesizing conducting inks that can replace interconnects and electrodes in devices. However, multifunctional devices require multilayers of semiconducting, conducting, insulating, and piezoelectric materials. Thus, there is a need to develop novel functional inks for printing functional devices.

This work was a step forward in preparing piezoelectric inks for inkjet printing employed for fabricating sensing devices. Polyvinylidene difluoride (PVDF), a thermoplastic polymer, was the material of choice due to its high chemical and mechanical stability and outstanding ferroelectric, piezoelectric, and pyroelectric properties in comparison with other organic materials [9]. These interesting properties lead to its wide application in electronic devices such as sensors, actuators, and capacitors [10,11]. PVDF can crystallize at five different phases of α, β, γ, δ, and ε based on the processing method; however, ferro-, piezo-, and pyroelectric properties of this polymer only stem from polar phases of β and γ. Several methods have been reported to increase the ratio of β-phase crystallinity to other non-polar phases, such as annealing [12], mechanical stretching [13], electrical poling [14], electrospinning [15], solvent casting [16], and addition of nucleating fillers [17]. So far, several fillers have been used to enhance the performance of PVDF films. Maity et al. [18] introduced molybdenum disulfide (MoS_2_) into PVDF using a polyaniline (PANI) interlinker. They reported that the incorporation of 10% filler led to 86% of β-phase crystallinity. Li et al. [19] enhanced the performance of PVDF-based nanogenerators by using ZnO nanorods as filler, coupled with electrospinning. The obtained membrane demonstrated promising open circuit voltage of ~85 V and short circuit current of ~2.2 μA. Pariy et al. [20] reported the incorporation of 0.1 w% reduced graphene oxide to enhance the piezoelectric constant (d_33_) of PVDF to 87 pm/V.

In this paper, we reported a method that uses BaTiO_3_ as nucleating filler to enhance β-phase crystallinity of PVDF for inkjet printing. Typical inkjet ink comprises four main components, namely (I) solvent, (II) functional particles, (III) binder, and (IV) additives. Solvent is used for dissolving binders and additives and tuning the final viscosity of ink. Furthermore, the solvent has crucial influence on the drying behavior of deposited droplets. The coffee-ring effect and slow/fast drying are typical problems associated with the improper selection of solvent. In this work, DMF (*N,N*-dimethylformamide) was used as the solvent of choice as it showed good solubility towards PVDF (with a dipole moment of 3.86 D) and promising surface wettability (γ = 37.1 mN/m) on a variety of substrates, including Kapton polyimide films. In the majority of inks, functional particles are the most crucial part of the ink, since they determine the final properties of printed patterns. In graphical inks, pigments are used as functional particles to endow different colors to printed images, while in functional inks the desired electrical properties such as conductivity, semi-conductivity, and piezoelectricity are provided by the particles (metal, carbon, ceramic, etc.). Here, BaTiO_3_ nanoparticles were used as functional particles to endow the ink with piezoelectric properties. BaTiO_3_ could also enhance polar-phase crystallinity of PVDF by acting as a nucleating agent, which resulted in enhancing the final piezoelectric performance of the obtained film. Polymeric binder is the other component of inkjet ink that brings about stable dispersion of functional particles and prevents their aggregation. PVDF is a polymer with good binding ability and demonstrates promising piezoelectric properties. Unlike metal inks, which require a sintering step to remove the binders, fabricated piezoelectric ink does not require any post-processing steps, since both the binder and the particles are piezoelectric, thus enabling printed devices on substrates with low thermal resistivity.

## 2. Experimental Section

### 2.1. Materials

PVDF pellets with average *M*_w_ = 275,000 and *M*_n_ = 107,000, DMF with purity of 99.8%, and BaTiO_3_ nanoparticles with an average size of 50 nm and purity of 99.9% were purchased from Sigma–Aldrich (St. Louis, MO, USA). All chemicals were used without further purifications.

### 2.2. Ink Preparation

To prepare PVDF-BaTiO_3_ nanocomposite inks, 5 g of PVDF was added to 95 g of DMF and magnetically stirred at 65 °C for 2 h until fully dissolved. Next, 1 g of BaTiO_3_ was added to 24 g of DMF and ball-milled for 1 h in a Retch planetary ball mill PM 100 (Haan, Germany) at the speed of 400 rpm with 50 g of 0.5 mm zirconia balls. To prepare inks with different concentrations, suitable amounts of PVDF solution and BaTiO_3_ dispersion were mixed and, if necessary, diluted with a suitable amount of DMF. Afterwards, the mixtures were placed in an ultrasonic bath for 15 min and then ball-milled at the speed of 300 rpm for 10 min.

### 2.3. Characterization

A Dimatix material printer DMP-2850, equipped with a drop-watcher and a fiducial camera, was used to print and study ejection behavior of the prepared inks. The fabricated inks were passed through Teflon filters with 2 µm pores and then injected into 1pL inkjet cartridges with a nozzle diameter of 9 µm. A capillary Cannon–Fenske (State College, PA, USA) viscometer tube was used to measure the static viscosity of the prepared inks at ambient temperature. A Theta Flex Optical Tensiometer (Gothenburg, Sweden) was used to measure the contact angle of the inks on microscope glass slides. Surface tension of the inks was calculated with contact angle data using Young’s equation:

γ_sv_ = γ_sl_ + γ_lv_·cosθ (1)

γ_sl_ is solid–liquid interfacial surface energy, γ_lv_ is liquid surface energy, and θ is the contact angle. Scanning electron microscopy (Zeiss Evo 10, Jena, Germany) was used to study the morphology and distribution of fillers in the printed films. To determine the crystalline structure of PVDF films, a powder X-ray diffraction spectroscopy technique (STOE fixed-stage powder diffractometer with a curved IP detector) was used. PerkinElmer (Waltham, MA, USA) Fourier transform infrared spectroscopy (FTIR) was used to determine the chemical structure and crystallinity of the printed films. A PerkinElmer DSC 8500 (Waltham, MA, USA) with an autosampler was used to investigate thermal behavior and the degree of crystallinity of the nanocomposite, according to the equation below:

(2) $$x_{c}=\;\frac{1}{x}\frac{\Delta H_{m}}{\Delta H_{\%100}}\times 100$$

where *x_c_* is the degree of crystallinity, *x* is the mass fraction of PVDF in the nanocomposite, Δ*H*_m_ is the melting enthalpy, and Δ*H*_%100_ is the melting enthalpy of a pure crystalline polymer, which is 104.6 J g^−1^ for PVDF [21]. Both heating and cooling measurements were carried out in the temperature range of 20–250 °C with a rate of 10 K min^−1^.

## 3. Results and Discussion

### 3.1. Determining Inkjet Printable Region

Drop ejection in a DOD inkjet printer is the result of consecutive deformation of piezoelectric ceramics in the printhead, which generates pressure pulses to push ink droplets out of the nozzles. Fluid mechanical properties determine jetting behavior and printability of inkjet inks. With fluids that are too viscous, the piezoelectric pulses cannot overcome viscous dissipation and the energy associated with forming a new surface, which leads to no ejection [8]. At the other end, with liquids that are too dilute, the pushing forces result in formation of two or more droplets (satellite drops), which can have deleterious effects on resolution and printability. In order to define the inkjet-printable region for the PVDF-BaTiO_3_ nanocomposite, six inks with a constant PVDF:BaTiO_3_ ratio but different concentrations were prepared, and their jetting behavior was observed at different cartridge temperatures. Table 1 summarizes the fluid mechanical properties of the fabricated inks and their jetting behavior at a constant trigger voltage of 30 V.

**Figure 1:**
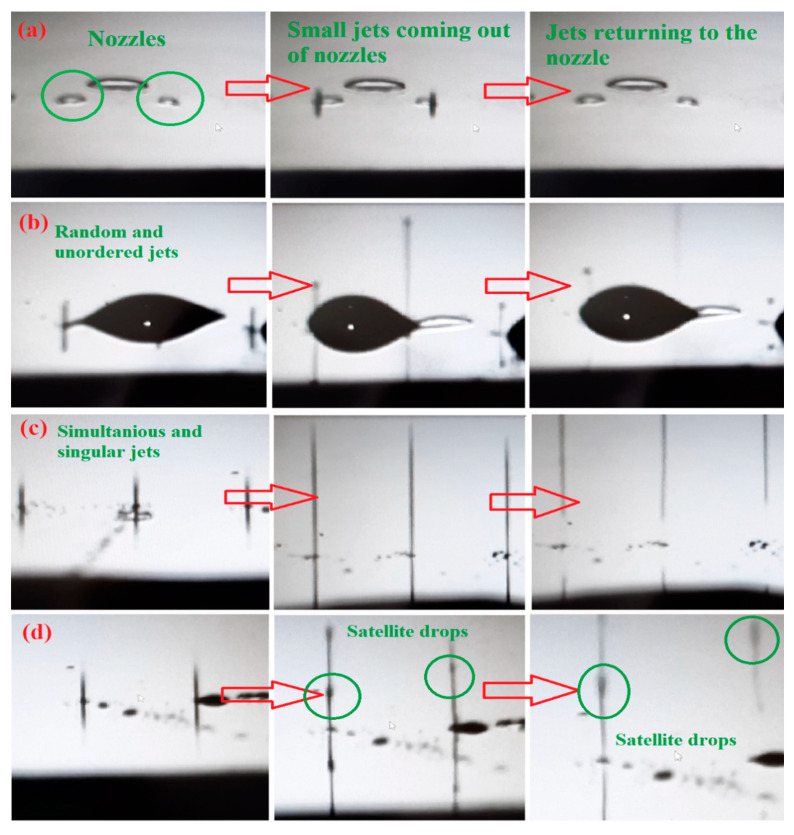
Jetting behavior of fabricated inks, (**a**) no ejection, (**b**) chaotic jet, (**c**) ideal jet, (**d**) satellite drops.

PB1 and PB2 inks with viscosities of 13.6 and 9.7 cP, surface tensions of 30.2 and 32.7 mN m^−1^, and corresponding Z values of 1.17 and 1.72, respectively, could not be ejected from the nozzle at any cartridge temperature (Figure 1a), indicating that the pushing pulses in the printhead could not overcome their viscous dissipation and supply sufficient energy to form a new surface in the form of a droplet. In ink PB3 with *Z* value of 2.79, it was observed that ink droplets were ejected from some nozzles in a completely haphazard way, yet other nozzles had no ejection. This ejection mode was named chaotic ejection and can be observed in Figure 1b. PB4 ink with *Z* value of 4.59 showed different ejection behaviors with changing cartridge temperature. At cartridge temperature of 30 °C the ejection was chaotic; however, when the temperature increased to 40 and 50 °C, ideal inkjet ejection was observed. Ideal inkjet ejection was characterized by consecutive and simultaneous ejection of droplets from nozzles in the form of singular drops (Figure 1c). Ideal ejection was also observed in PB5 ink with *Z* value of 8.23 at all three cartridge temperatures. In ink PB6 with *Z* value of 13.56, the ejection mode was in the form of satellite drops (Figure 1d). Satellite drops, also known as secondary drops, are the extra droplets formed during the detaching of the main droplet from the nozzle. This ejection behavior is observed when the ink is too dilute and piezoelectric pulses in the printhead can produce more separate surfaces from bulk ink. In summary, the interval of 4.59 < *Z* < 13.56 was obtained as the inkjet-printable range for PVDF-BaTiO_3_ nanocomposite inks. Inks with lower *Z* value could not be ejected from nozzles, and inks with *Z* value above this range formed satellite drops during ejection.

**Figure 2:**
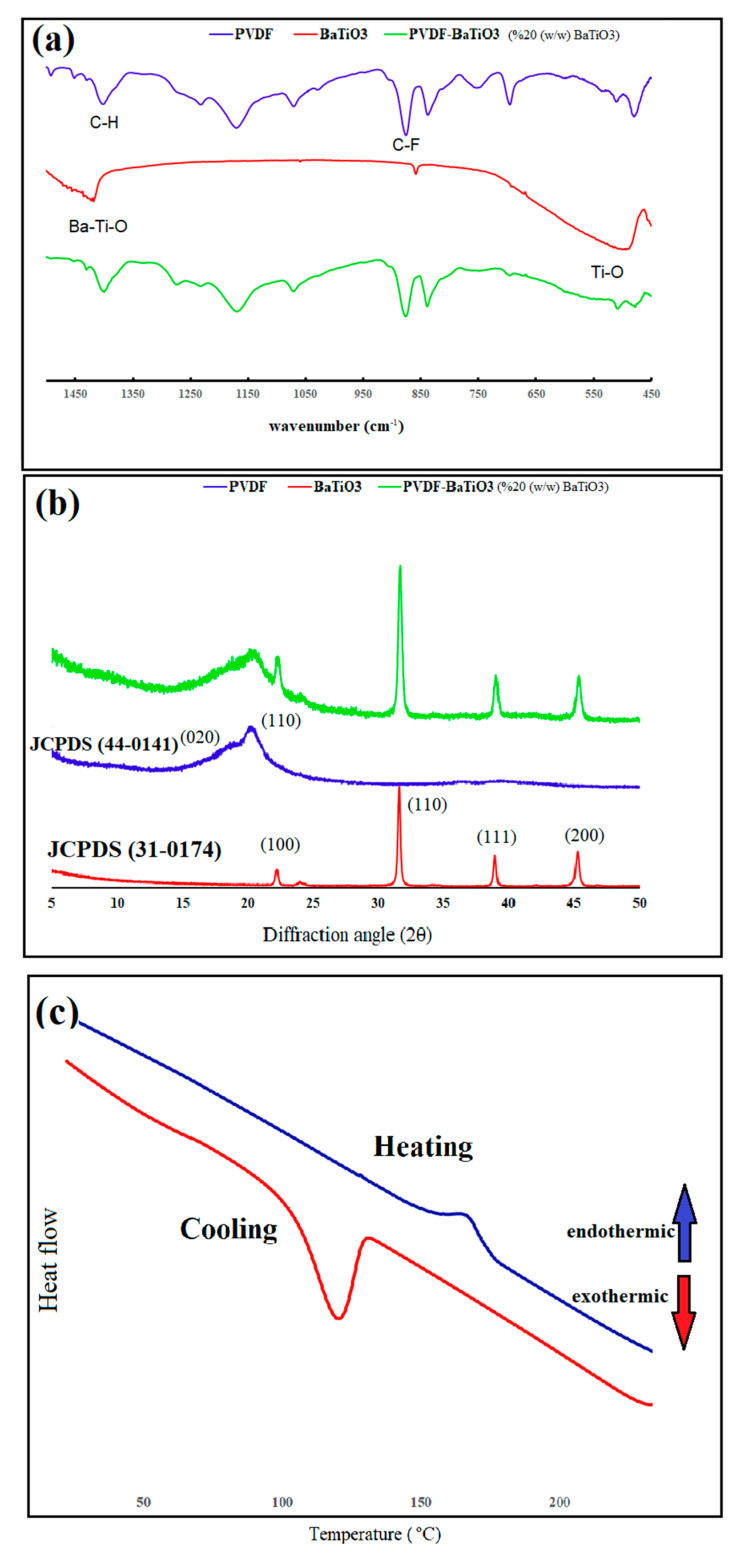
(**a**) FT-IR and (**b**) XRD spectra of polyvinylidene difluoride (PVDF), BaTiO3, and PVDF-BaTiO_3_ nanocomposite, (**c**) DSC thermogram of the nanocomposite with 20% BaTiO_3_ content.

In order to confirm the chemical structure of the printed films, FT-IR and XRD spectroscopy were employed. Figure 2a demonstrates the FT-IR spectra of PVDF, BaTiO_3_, and their printed nanocomposite. In PVDF, the absorption peaked at 1231, 1266, and 1401 cm^−1^, which was attributed to the wagging vibration of CH_2_; the peak at 836 cm^−1^ originated from C–C–C asymmetric stretching, and absorption bands at 874 cm^−1^ were attributed to C–F vibrations [22,23,24]. Different crystalline phases of PVDF could also be determined using the FT-IR technique. Peaks around 480, 532, 760, 795, 1149, 1209, and 1423 cm^−1^ could be assigned to α-phase, while peaks attributed to β-phase appeared at 473, 840, and 1266 cm^−1^ [25,26,27,28]. BaTiO_3_ was characterized by two main absorption peaks, one at around 492 cm^−1^, which was attributed to Ti–O vibrations, and the other at 1440 cm^−1,^ related to Ba–Ti–O stretching vibrations [29]. As seen in Figure 2a, the FT-IR spectrum of PVDF-BaTiO_3_ nanocomposite possessed all the characteristic peaks of both materials. Although the characteristic peaks of PVDF and BaTiO_3_ overlapped at 490 and 1440 cm^−1^, the broad absorption bands of BaTiO_3_ were distinguishable within the sharp peaks of PVDF.

**Figure 3:**
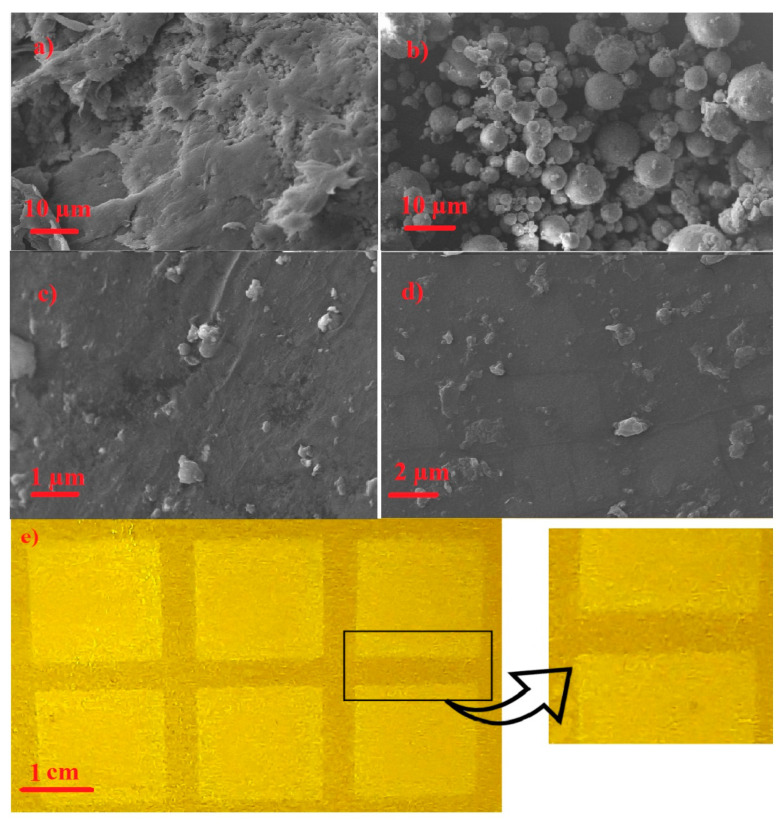
SEM images of (**a**) PVDF, (**b**) BaTiO_3_, (**c**) and (**d**) PVDF-BaTiO_3_ (%20 (*w*/*w*) BaTiO_3_) nanocomposite at different magnifications. (**e**) Image of the printed PVDF-BaTiO_3_ (%20 (*w*/*w*) BaTiO_3_) inkjet ink on a Kapton substrate.

To further confirm the chemical structure of the printed films, XRD measurements were carried out on a pure PVDF film, BaTiO_3_, and the nanocomposite (Figure 2b). PVDF showed a broad XRD peak starting at 2θ = 15.5° and finishing at 2θ = 21.7°. This broad peak was composed of two separate peaks: one at around 18.7°, corresponding to the α crystalline phase, and the other at 20.5°, attributed to the β-phase crystalline structure of PVDF. The XRD pattern of BaTiO_3_ showed four sharp peaks in the diffraction angle interval of 5° < 2θ < 50°. The peaks were located at diffraction angles of 22.2°, 31.5°, 38.9°, and 45.3°, which were indexed (100), (110), (111), and (200), respectively. No split in the peak of (200) at 2θ = 45.3° indicated cubic crystalline structure of nano-BaTiO_3_ powders. The XRD spectrum of the printed PVDF-BaTiO_3_ nanocomposite possessed all the characteristic peaks of both products, confirming the predicted structure of the obtained film. Crystallite size of PVDF could also be calculated from the XRD spectra using Scherrer´s equation [30]. β-phase crystallite size of the nanocomposite was 63.6 nm, which was smaller than that of pure PVDF (76.3 nm). Heating and cooling DSC measurements were also carried out to investigate melting temperature and degree of crystallinity of the nanocomposite with 20% of BaTiO_3_. In the endothermic process, the melting peak appeared around 166 °C, and the degree of crystallinity was 43.7%, according to Equation (2). The glass transition temperature (*T*_g_) of PVDF was around −35 °C, so the corresponding peak did not appear in the obtained thermogram. SEM was used to investigate the morphology of the obtained nanocomposite. Figure 3a demonstrates the surface structure of PVDF, which consisted of both amorphous regions and spherulite phases. Figure 3b shows BaTiO_3_ before the wet milling process, which indicated highly aggregated particles with average diameter of around 5 µm. The printed PVDF-BaTiO_3_ film (Figure 3c,d) was homogeneous and showed uniform morphology. The nanocomposite film did not show agglomerated particles, as evident before milling. Furthermore, no sign of voids and micro cracks was observed in the surface of the nanocomposite, confirming that inkjet printing could be employed for preparation of uniform PVDF-BaTiO_3_ films. Figure 3e is the image of patterns printed on a Kapton substrate.

### 3.2. Influence of BaTiO_3_ Content on β-Phase Crystallinity

The unit cell of α-phase PVDF consists of chains with trans-gauche-trans-gauche (TGTG) conformation and total lattice energy of −25.23 kJ mol^−1^, while β-phase structure had all-trans conformation (TTTT) with total lattice energy of −23.97 kJ mol^−1^ [31]. As nature prefers lower energy states, α-phase should be the dominant polymorph of PVDF. However, there are several strategies to enhance the ratio of β- to α-phase. For instance, when transforming bulk PVDF to thin films through printing or solution casting methods, polymer chains tend to attain more parallel structures, which results in increasing the β-phase ratio.

**Figure 4:**
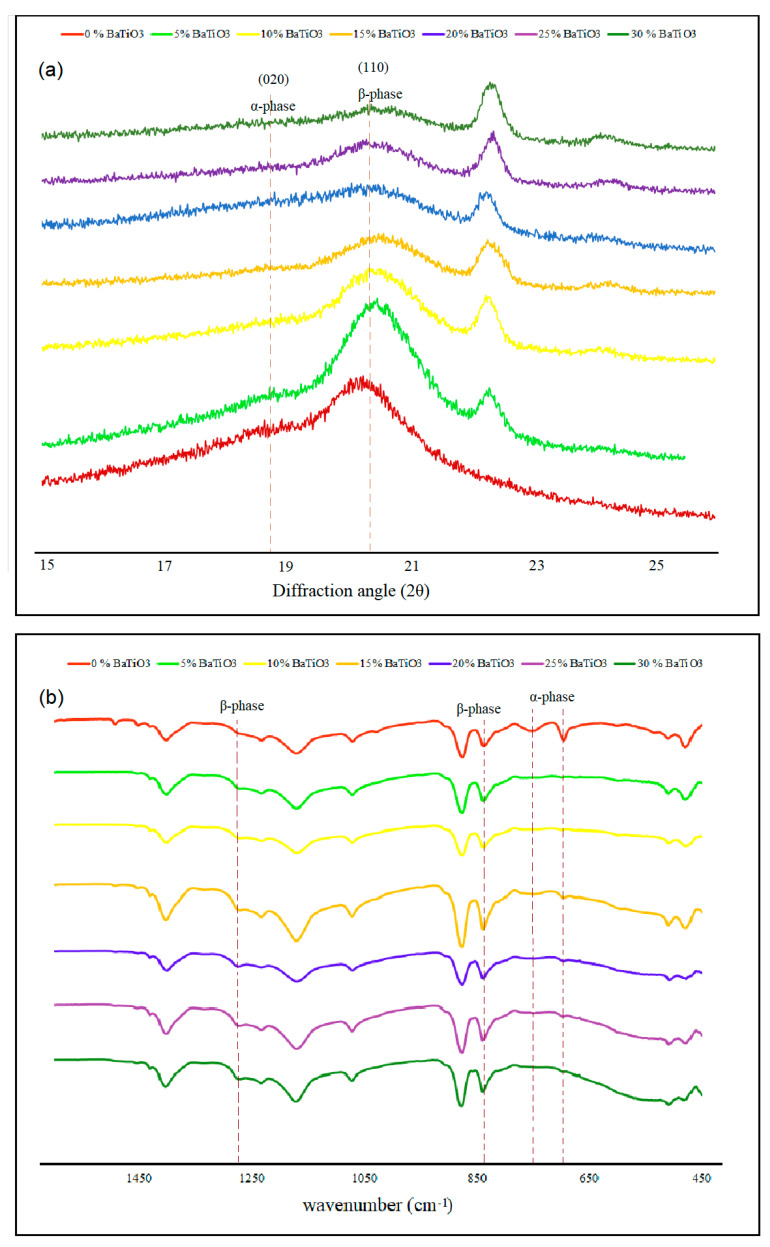
(**a**) XRD and (**b**) FT-IR spectra of PVDF nanocomposites with different BaTiO_3_ content.

**Figure 5:**
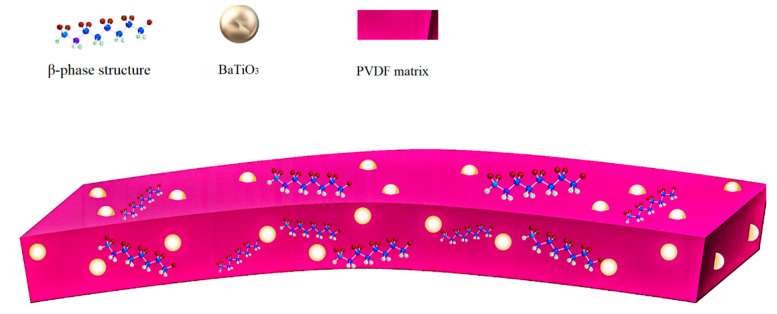
Schematic of the nucleating effect of BaTiO_3_ nanoparticles in PVDF.

Addition of nucleating fillers was the approach taken in this work to enhance the ratio of polar to non-polar phases. To investigate the influence of BaTiO_3_ nanofillers on β-phase crystallinity of PVDF, inks with different concentrations of BaTiO_3_ (0%, 5%, 10%, 15%, 20%, 25%, 30%) were prepared and inkjet-printed. Figure 4a shows the XRD spectra of the printed films in the diffraction angle interval of 15° < 2θ < 25°. The spectra showed two distinct peaks at 18.7°, corresponding to the (020) reflection of α-phase, and 20.6° representing the (110) plane diffraction peak of the β-phase lattice. According to Figure 4a, the film containing 5% BaTiO_3_ had higher (110) and lower (020) intensity of peaks in comparison with those of pure PVDF, thus confirming β-phase enhancement through α-to-β transformation. By increasing the ratio of BaTiO_3_, the intensity of both (110) and (020) peaks decreased progressively, indicating that more filler content deteriorated the overall crystallinity of PVDF and led to more amorphous structures. FT-IR spectroscopy was used to further corroborate the obtained XRD results. Characteristic absorption bands of the α-phase appeared around 410, 489, 532, 614, 763, 795, 854, 975, 1149, 1209, 1383, and 1423 cm^−1^, whereas characteristic bands of β-phase were around 445, 473, 840, 1275, and 1431 cm^−1^ [28]. According to Figure 4b, the intensity of the peak at 763 cm^−1^, representing the α-phase, decreased remarkably in the spectrum of the sample with 5% BaTiO_3_, in comparison with that pure of PVDF, while the absorption band at 840 cm^−1^, corresponding to the β-phase, was strengthened. Figure 5 shows how BaTiO_3_ nanoparticles worked as nucleating spots for growing the β crystalline phase in PVDF. In summary, crystallization occurs in two stages: nucleation and growth. The free energy of crystallization is the sum of (I) free energy for formation of a stable nucleus embryo (Δ*G*°) and (II) free energy of polymer chain diffusion to join the growing crystals (Δ*G*_η_) [32]. The presence of nucleating fillers significantly decreases Δ*G*°, leading to more overall crystallinity. The domination of the β-phase in comparison to other polymorphs can be attributed to its all-trans (TTTT) configuration, where negative dipoles (C–F bonds) are in one direction and positive dipoles (C–H bonds) on the other side. As BaTiO_3_ particles possess positive surface charge, they tend to absorb negative dipoles; hence, β-phase crystals, which have the highest dipole moment among all the phases, tend to grow and dominate on the surface [33].

## 4. Conclusions

PVDF and its copolymers are promising electroactive organic materials, which demonstrate remarkable piezoelectric properties. PVDF can crystallize at five different phases of α, β, γ, δ, and ε, while piezoelectric properties only originate from the polar phases of β and γ. This work employed the addition of nucleating fillers to enhance the ratio of polar to non-polar phases in PVDF. We developed inkjet-printable PVDF-BaTiO_3_ inks with enhanced β-phase crystalline structure through the incorporation of 5 w% BaTiO_3_. The ink was printed successfully and made a homogeneous thin film without observable voids and microcracks. The showcased work is a step forward in building a library of functional materials for printed electronic applications.

## Figures and Tables

**Table 1:** Properties of fabricated inks and their jetting behavior at a constant trigger voltage of 30 V.

Sample Name	PVDF Concentration (mg mL^−1^)	BaTiO_3_ Concentration (mg mL^−1^)	Density (kg m^−3^)	Viscosity (cP) (at Room Temperature)	Surface Tension (mN m^−1^)	Printhead Temperature(s) (°C)	*Z* Value	Jetting Behavior
**PB1**	40	8.0	972	13.6	30.2	30, 40, 50	1.17	No ejection
**PB2**	32	6.4	967	9.7	31.7	30, 40, 50	1.72	No ejection
**PB3**	24	4.8	963	6.0	32.4	30, 40, 50	2.79	Chaotic jet
**PB4**	16	3.2	959	3.7	33.5	30	4.59	Chaotic jet
**PB4**	16	3.2	959	3.7	33.5	40, 50	4.59	Ideal jet
**PB5**	8	1.6	955	2.1	34.8	30, 40, 50	8.23	Ideal jet
**PB6**	1	0.2	959	1.3	36.0	30, 40, 50	13.56	Satellite drop
